# 

**Published:** 1902-03-29

**Authors:** 


### NOTICE TO CORRESPONDENTS.

All MSS., Letters, Books for Review, nnd other matters intended for the Editor should be addressed The Editor "The Hospital," The Hospital Building, 28 & 29 Southampton Street, Strand, W.C.

The Editor cannot undertake to return rejected MS., even when accompanied by stamped directed envelope.

### Notice to Advertisers and Subscribers.

All Advertisements, Orders for Copies of The Hospital and Business Communications should be addressed to **THE MANAGER, (Not to the Editor)**, The Hospital, 28 & 29 Southampton Street, Strand, London, W.C.

The Cost of Subscription, post free, is as follows.—Per week, 2½d.; per quarter, 2s. 8d; per half-year, 5s. 3d.; per annum, 10s. 6d. Foreign:—Per annum, 15s. 2d., payable, in all cases, in advance.

**NOTICE.—Vol. XXX. of "The Hospital" (April 1901, to September 1901), in handsome cloth binding can now be obtained at the office of this Journal, price, 7s. 6d. Cases for binding the Numbers contained in this Volume 1s. 6d. Index to Vol. XXX., 2½d., post free.**

**The Monthly Part for April will be ready on April 1st. Price 6d., by post 8d.**

### SCRAPS AND GLEANINGS.

The committee of the Southend Victoria Hospital announce the receipt of a legacy of £2,700 odd, from the estate of the late Mr. J. G. Lawrence. This sum is to go to the Endowment Fund, and is the first contribution which this fund has received.

The number of patients treated in the Royal Southern Hospital (Liverpool), during last year was 12,964, of which 2,351 were in-patients and 10,613 out-patients. In the ward devoted to patients suffering from tropical diseases there had been treated 130 cases.

The committee of the Hospital Sunday and Saturday Fund of Manchester state that the medical charities of Manchester and Salford require about £12,000 to meet the deficiency of income for 12 months as shown by the last published accounts, and appeal to the public for increased contributions.

### THE STUDENT OF MEDICINE.

### APPOINTMENTS

**H. T. D. Acland**, F.R.C.S.Eng., appointed Medical Officer to St. Thomas's Home, St. Thomas's Hospital. **Theodore Ambrose**, M.B., appointed Resident Medical Officer to the Sydney Hospital, New South Wales. **D. H. Beegling**, B.A.Sydney Univ., M.B., C.M.Edin., appointed Medical Officer to the Post Office at Huddersfield. **Richard R. Cruise**, M.R.C.S.Eng., L.R.C.P.Lond., appointed House Surgeon to the Bristol Eye Hospital. **J. H. R. Garson**, M.B., C.M.Edin., appointed District Medical Officer of the Chester-le-Street Union. **Arthur Greene**, B.A., M.D., L.M.etc.Dubl., appointed Clinical Assistant to the Royal Westminster Ophthalmic Hospital. **J. E. Judson**, M.R.C.S.Eng., L.R.C.P.Lond., late Senior Resident Medical Officer of the Manchester Workhouse, Crumpsall, appointed House Surgeon to the District Infirmary, Ashton-under-Lyne. **Middleton Martin**, M.B., B.C., D.P.H.Camb., appointed Medical Officer of Health for the Stroud Urban District Council. **J. G. McNaughton**, M.D., M.R.C.P.Edin., appointed Honorary Medical Officer to the Chorlton-upon-Medlock Dispensary, Manchester. **G. Norman Meachen**, M.B., B.S.Lond., M.R.C P.Edin., appointed Honorary Dermatologist to the Tottenham Hospital, **N. Keith Monsarrat**, M.B., F.R.C.S.E., appointed Honorary Surgeon to the David Lewis Northern Hospital, Liverpool. **R. Morison**, M.B., Ch.B., appointed Junior House Surgeon to the Birkenhead Borough Hospital. **A. Muscio**, M.B., appointed Resident Medical Officer to the Prince Alfred Hospital, Sydney, New South Wales. **C. Nesbit**, M.B., B.Ch., R.U.I., appointed Certifying Factory Surgeon for the Rondalstown District of the County of Antrim. **William Seldon**, M.B., appointed Resident Medical Officer to the Sydney Hospital, New South Wales. **G. Morgan Soper**, M.R.C.S., L.R.C.P., appointed Assistant House Surgeon to the Royal Albert Hospital and Eye Infirmary, Devonport. **D. Wallace**, M.B., appointed Resident Medical Officer to the Prince Alfred Hospital, Sydney, New South Wales. **G. F. White**, M.D., appointed Senior House Surgeon to the Birkenhead Borough Hospital. **W. Cheyne Wilson**, M.D.Edin., appointed Physician to the South Devon and East Cornwall Hospital.

